# Anomalous Terminal Shear Viscosity Behavior of Polycarbonate Nanocomposites Containing Grafted Nanosilica Particles

**DOI:** 10.3390/nano11071839

**Published:** 2021-07-15

**Authors:** Vaidyanath Ramakrishnan, Johannes G. P. Goossens, Theodorus L. Hoeks, Gerrit W. M. Peters

**Affiliations:** 1 Department of Mechanical Engineering, Eindhoven University of Technology, P.O. Box 513, 5600 MB Eindhoven, The Netherlands; g.w.m.peters@tue.nl; 2 Department of Chemical Engineering and Chemistry, Eindhoven University of Technology, P.O. Box 513, 5600 MB Eindhoven, The Netherlands; theo.hoeks@sabic.com

**Keywords:** nanocomposites, viscosity control, tube diameter, disentanglement, entanglement density, grafting, rheology, differential scanning calorimetry

## Abstract

Viscosity controls an important issue in polymer processing. This paper reports on the terminal viscosity behavior of a polymer melt containing grafted nanosilica particles. The melt viscosity behavior of the nanocomposites was found to depend on the interaction between the polymer matrix and the nanoparticle surface. In the case of polycarbonate (PC) nanocomposites, the viscosity decreases by approximately 25% at concentrations below 0.7 vol% of nanosilica, followed by an increase at higher concentrations. Chemical analysis shows that the decrease in viscosity can be attributed to in situ grafting of PC on the nanosilica surface, leading to a lower entanglement density around the nanoparticle. The thickness of the graft layer was found to be of the order of the tube diameter, with the disentangled zone being approximately equal to the radius of gyration ($R_{g}$) polymer chain. Furthermore, it is shown that the grafting has an effect on the motion of the PC chains at all timescales. Finally, the viscosity behavior in the PC nanocomposites was found to be independent of the molar mass of PC. The PC data are compared with polystyrene nanocomposites, for which the interaction between the polymer and nanoparticles is absent. The results outlined in this paper can be utilized for applications with low shear processing conditions, e.g., rotomolding, 3D printing, and multilayer co-extrusion.

## 1. Introduction

Advancements in technology have led to the development of new materials for a gamut of applications. Composites have a special place in this race, as the combination of two or more materials—often with very different constituent characteristics—leads to a product with unique properties. Therefore, it is not surprising that a lot of research has focused on this class of materials. In the last two decades, composites with nanofillers have gained considerable attention. The advantage of using polymer nanocomposites is that the macroscopic properties can be drastically enhanced with low concentrations of nanofiller (<5 wt%) due to its large specific surface area. Changes in melt rheology, a higher heat distortion temperature and a low coefficient of thermal expansion (CTE) are some of the macroscopic features that can be tuned by choosing the appropriate type of nanofillers [1,2,3,4]. However, in order to achieve the desired properties in nanocomposites, it is of utmost importance to control the filler dispersion in the polymer matrix and to understand the underlying physics that lead to these changes. With this in mind, this paper focuses on rheological properties of melt-compounded polycarbonate (PC)/silica nanocomposites and the relation of these properties to the physics at the nano-particle level.

Polycarbonate (PC) is an important engineering plastic that is used both as filled and unfilled in applications, such as automotive, lenses, building and construction, due to the combination of toughness, transparency and heat resistance [5]. One of the concerns of using polycarbonate is that its high viscosity might prevent molding parts that are very thin with low in-mold stresses. Therefore, strategies to reduce the processing viscosity would widen the application space of PC. It has been shown in many studies that the addition of fillers to a polymer matrix can both increase or lower its viscosity and provide reinforcement [2,6,7,8,9,10,11,12,13,14,15,16,17,18,19]. While the increase is understood, the decrease has been attributed to changes in free volume (for the polymer radius of gyration < filler size) or adsorption of high molar mass chains onto the nanoparticle surface [16,18].

Previous work on PC composites showed that blending PC with inorganic particles, such as hollow glass beads, barium sulfate (${\mathrm{BaSO}}_{4}$), calcium carbonate (${\mathrm{CaCO}}_{3}$) or nanosilica, results in different viscosity behavior [20]. Chen et al. showed that the addition of hollow glass beads to PC lowered the melt viscosity by over a decade at 30% glass beads loadings [21], while Wang et al. reported a drop in the apparent shear viscosity and a concomitant improvement of the tensile modulus by the addition of 1 wt% ${\mathrm{CaCO}}_{3}$ to PC [22]. Increasing the concentration of ${\mathrm{CaCO}}_{3}$ resulted in a continuous decrease 50% in the viscosity and glass transition temperature, $T_{g}$, (by 6 °C), which was found to be equivalent to increasing the processing temperature by approximately 10 °C. A similar observation was also made in the case of PC/multiwall carbon nanotube (MWCNT) composites by Jin et al., where the decrease in viscosity was attributed to an increase in the mobility of the PC molecules, due to an increase in free volume [23]. Their hypothesis was supported by a decrease in $T_{g}$ of the nanocomposites compared to neat PC. All the above studies in PC composites attributed the viscosity drop to either polymer chain disentanglement due to a rotating spheres (ball bearing) effect or the excluded free volume at the solid particle-melt interface, but did not consider other causes, such as plasticization or molar mass changes. This paper attempts to explain the interesting rheological properties of melt-compounded polycarbonate/silica nanocomposites in light of the work done previously on using nanoparticles for reducing viscosity. Polycarbonates of various molar masses with a constant poly-dispersity index (PDI) are used. Furthermore, the results are compared against poly(styrene)/silica nanocomposites, as PS and PC have a different interaction with the nanoparticles. The nanocomposites were characterized using rheological, thermal and chemical techniques, as discussed below.

## 2. Theoretical Background

### 2.1. Viscosity Behaviour

It is known that the rheological properties are affected by the addition of (nano)filler. Considerable experimental and theoretical studies showed that the viscosity of a particulate suspension increases with particle volume fraction [24]. Therefore, any property, such as the complex viscosity ($\eta^{*}$) or the shear modulus (G), of a (nano)composite is given by property=property(with no filler)×f(ϕ), where f(ϕ) is a function dependent on the filler concentration [25,26]. The properties, for example, viscosity (η), are expressed as a polynomial function, as given below, where ϕ is the particle volume fraction:

(1) $$\frac{\eta (\phi )}{\eta (\phi =0)}=\left(1+\left[\eta \right]\phi +a^{2}\phi^{2}+a^{3}\phi^{3}+\cdots \right)$$

where,

[η] = 2.5; $a^{2}$, $a^{3}$, ... $a^{n}$ = 0 for dilute solutions (Einstein relation) [27].
[η] = 2.5; $a^{2}$ = 14.4 for concentrated solutions (Guth–Gold relation) [28,29].

The theory discussed above is limited to perfect hard spheres in a Newtonian medium and is valid when radius(filler) > radius(solvent molecule) or $r_{particle}$ > $r_{medium}$ but cannot be strictly used for nanocomposites. The above theory is not applicable in the case of polymer nanocomposites, as the ratio of the radius of the polymer chain and particle is order 1, i.e., $R_{g}$/$r_{particle}$> 1, where $R_{g}$ is the radius of the gyration of the polymer chain, and the presence of enthalpic interactions between filler and polymer. Consequently, particle aggregation, chain adsorption, grafting and slip need to be taken into consideration. It is important to state here that Equation (1) has been validated for micron-sized fillers and not so much for polymer nanocomposites [2,30]. These observations indicate that the polymer–particle, particle–particle interactions and entanglement number are important parameters, and confinement and surface effects provided by the large specific surface area of nanoparticles could lead to conformational changes of polymer molecules, which affect the viscosity around the nanoparticles and, concomitantly, the viscosity of the bulk.

### 2.2. Definition of Rheological Properties

In this article, we use the continuous relaxation spectrum and the steady state compliance to provide insights into the micro-structure of the polymer nanocomposite. This was calculated using following:

(2) $$G^{\prime }\left(\omega \right)=\int_{-\infty }^{\infty }H\left(ln\tau \right)\frac{\omega^{2}\tau^{2}}{1+\omega^{2}\tau^{2}}dln\tau$$

(3) $$G^{\prime \prime }\left(\omega \right)=\int_{-\infty }^{\infty }H\left(ln\tau \right)\frac{\omega \tau }{1+\omega^{2}\tau^{2}}dln\tau$$

(4) $$J^{\prime }\left(\omega \right)=\frac{\left(1/G^{\prime }\left(\omega \right)\right)}{1-tan^{2}\delta }$$

(5) $$J^{\prime \prime }\left(\omega \right)=\left(1/G^{\prime }\left(\omega \right)\right)\times \left(1-tan^{2}\delta \right)$$

where δ is the phase angle, H(lnτ) is the continuous relaxation function and τ is the relaxation time.

The plateau $G_{N0}$ [31] and steady state compliance modulus ($J_{0}$) are given by:

(6) $$G_{N0}=G^{\prime }{\left(\omega \right)}_{tan\delta \to minimum}$$

(7) $$J_{0}=J^{\prime }{\left(\omega \right)}_{\omega \to 0}$$

### 2.3. Definition of Thermal Properties

The unique properties of nanocomposites are related to the modification of the structure and dynamics around and at the particle surface. In order to probe these nanoscale effects, i.e., changes in molecular dynamics, the $T_{g}$ and heat capacity are often used as a probe. Lipatov and Privalko [32] recognized the importance of measuring and analyzing the absolute $C_{p}$ in order to understand polymer dynamics. The $C_{p}$ changes in chain mobility caused by the nanoparticles do not extend throughout the matrix, but only a few nanometers around the particle. The existence of such a layer was shown for several nanocomposites [33,34,35,36,37,38,39,40]. In order to characterize this layer, we make use of the concept of the rigid amorphous fraction (RAF) introduced by Menczel and Wunderlich [41] for semi-crystalline polymers extended to polymer nanocomposites by Sargsyan et al. [42] and Wurm et al. [43]. In the case of polymer nanocomposites, this relationship can be written as

(8) $$RAF=1-\phi -\left(\frac{\Delta C_{p}}{\Delta C_{p,pure}}\right)$$

where $\Delta C_{p}$ and $\Delta C_{p,pure}$ are the heat capacity increments at the $T_{g}$ for the nanocomposite and the 100% amorphous polymer, respectively. This is most commonly measured using calorimetry (DSC, TM-DSC). It is important to note that the above relation is valid, provided that the ratio between the filler and the RAF remains constant. For high filler loadings, the ratio might decrease due to filler agglomeration. This indicates that not all nanoparticles are covered with the same amount of RAF. In such cases, in order to have a direct comparison of the $C_{p}$ of the polymer fraction of the nanocomposite, the contribution of the filler has to be subtracted. For the polymer fraction of the nanocomposites, Equation (8) can be rewritten for the immobilized fraction of the polymer:

(9) $$RAF_{polymer}=1-\left(\frac{\Delta C_{p}}{\Delta C_{p,pure}}\right)$$

## 3. Materials and Methods

The bisphenol A-based poly(carbonate) (PC) and poly(styrene) (PS) used in this study were provided by SABIC’s Innovative Plastics business, Bergen op Zoom, The Netherlands. The PCs used are coded as PCxy, where x stands for the weight-average molar mass ($M_{w}$) and y is the vol% of nanosilica. The silica nanoparticles, with an average particle diameter of 12 nm ± 3 nm, were purchased from Nissan Chemical Industries, Japan, as a suspension in methyl ethyl ketone (MEK) with approx. 30 wt% nanoparticles. All materials were used as received. Table 1 summarizes the characteristics of the polymers, including molar mass, polydispersity (PDI), tube diameter ($R_{e}$) and the radius of gyration ($R_{g}$) obtained from literature. These were measured by using size exclusion chromatography (SEC) as described below.

### 3.1. Preparation of Nanocomposites and Samples for Testing

The polymer powders were dried for 12 h at 110 °C (60 °C for PS) before mixing with the nanosilica dispersion at different concentrations (0.5–5 vol%) and 0.1 wt% tris(2,4-di-tert-butylphenyl) phosphite as processing stabilizer. The material was then dried for 24 h to remove the solvent. The material was compounded by using a ZSK-25 twin-screw extruder (Krupp Werner and Pfleiderer, GmbH, Dusseldorf, Germany) at 300 °C (200 °C for PS) and a screw speed of 300 rpm. The pellets from the extruder were remixed using a home-built, recirculating, twin-screw mini-extruder (internal volume of 5 ${\mathrm{cm}}^{3}$) with a screw speed of 75 rpm for 15 min under $N_{2}$ atmosphere to ensure that the nanoparticles are well dispersed. After drying the pellets of PC/silica and PS/silica nanocomposites, sheets with a thickness of 1.0 mm and 0.5 mm were prepared by using compression molding at 250 °C for 10 min at a pressure of 50 bars. Samples for the rheological characterization (25 and 8 mm in diameter, 1.0 and 0.5 mm in thickness) were cut from these sheets.

#### Sample Preparation for FTIR and XPS

A procedure similar to the one described by Wang et al. was used for extracting the nanosilica from the nanocomposites [45]. The method is discussed in detail in our previous paper [46].

### 3.2. Characterization of Samples

#### 3.2.1. Transmission Electron Microscopy (TEM)

Morphological studies were performed by using a Tecnai G2 transmission electron microscope, operated at 120 kV in bright field. Ultra-thin sections of 100 nm were obtained at room temperature by using a Leica Ultracut E microtome. Staining of the sections was not required, since the electron density of silica is much higher than that of PC and PS. The silica nanoparticle size and the particle size distribution were determined by using MATLAB (image analysis toolbox). A total of 20 images were analyzed using adaptive thresholding, followed by edge detection and mapping the pixel to get accurate results.

#### 3.2.2. Size Exclusion Chromatography (SEC)

The molar mass and polydispersity index of PC was determined by using SEC on a Polymer Laboratories PL gel 5 μm MiniMIX-C 250 × 4.6 mm column and a UV detector, operated at 254 nm. The method used is discussed in [46]. The molar mass change was found to be negligible before and after testing and compounding(shown in the supplementary information, Table S1).

#### 3.2.3. Melt Rheology

The oscillatory rheology of the PC and PS nanocomposites was measured using an ARES-G2 rheometer at temperatures ranging from 170 to 300 °C using both the 8 and 25 mm parallel plate geometry under a $N_{2}$ blanket. Prior to measuring all the samples were dried at 120 °C for 4 h in a vacuum oven. The samples were loaded at 120 °C and heated to the desired temperature. The gap between the parallel plates was adjusted to a final gap of approx. 1.0 mm. Strain sweeps were conducted at several frequencies to identify the maximum strain for testing in the linear viscoelastic range. Frequency sweeps were carried out from ω = 0.1 to 500 rad/s and mastercurves were constructed at a reference temperature of 250 °C using the Time-Temperature Superposition (TTS) principle. The horizontal shift factor ($a_{T}$) is given by the WLF relationship as [47]:

(10) $$a_{T}=\frac{C_{1}\left(T-T_{0}\right)}{C_{2}\left(T-T_{0}\right)}$$

here, $C_{1}$ and $C_{2}$ are empirical constants and $T_{0}$ is the reference temperature and T is the test temperature. The viscosity values reported correspond to the complex viscosity at 1 rad/s for PC and 2 rad/s for PS. No viscosity models were used to calculate the zero-shear viscosity, as all the composites, both PC and PS nanocomposites, did not follow the Cox–Merz relation [48] (see supplementary information, Figures S2 and S3). This was shown to be true in other nanocomposites, including PC/silica nanocomposites [18,49,50,51,52]. We would like to note here that other potential effects, such as wall slip, in-homogeneous flow and molar mass degradation (during preparation and measurements), were accounted for (see supplementary information, Tables S1 and S2, and Figure S2) [7,53,54,55,56,57].

#### 3.2.4. Differential Scanning Calorimetry (DSC)

The glass transition temperature ($T_{g}$), heat capacity and enthalpy change curves were determined by a Q2000 DSC (TA Instruments). The instrument was calibrated using Indium for temperature and enthalpy, and sapphire for heat capacity. The samples used for all measurements were cut from compression-molded disks of 0.5 mm; the diameter of each disk was 4.0 mm. This was done to ensure good thermal contact with the sample and the dimensions matched that of the calibration sample. All DSC tests were performed after the thermal history was erased. This was done by heating the sample to 200 °C, followed by cooling at 10 °C/min. The $T_{g}$ was measured by heating the sample from 30 to 200 °C at 10 °C/min using a sample mass between 3–5 mg. Heat capacity measurements were performed by using temperature-modulated DSC from 30 to 200 °C. The measurements were performed using 10–15 mg of sample with a heating rate of 2 °C/min with a modulation amplitude of 1 °C and with a period of 120 s. The error in the heat capacity measurements was estimated to be approximately 3%. The $T_{g}$ was calculated as the mid-point of the heat capacity step.

#### 3.2.5. X-ray Photoelectron Spectroscopy (XPS)

XPS studies were carried out on a Kratos Axis Ultra DLD spectrometer equipped with a monochromatic Al Kα X-ray source with an energy of 1486.6 eV, i.e., hν = 1486.6 eV, operated at 150 W, a multi-channel plate and delay line detector under $1.0\times 10^{-9}$ Torr vacuum. Survey and high-resolution spectra were collected at fixed analyzer pass energies of 160 and 20 eV, respectively. The samples were mounted in floating mode in order to avoid differential charging. Charge neutralization was required for all samples. The binding energies were referenced to the C1s (C-C) binding energy and was set at 284.8 eV.

#### 3.2.6. Fourier Transform Infrared (FTIR) Spectroscopy

The FTIR method used in this work is mentioned in detail in our previous paper [46].

## 4. Results and Discussion

In this section, the state of dispersion of the silica particles in both the PC and the PS nanocomposites is presented, followed by the rheological behavior of the nanocomposites. The possible mechanisms for the behaviors observed are addressed in the last section.

### 4.1. State of Dispersion

**Figure 1:**
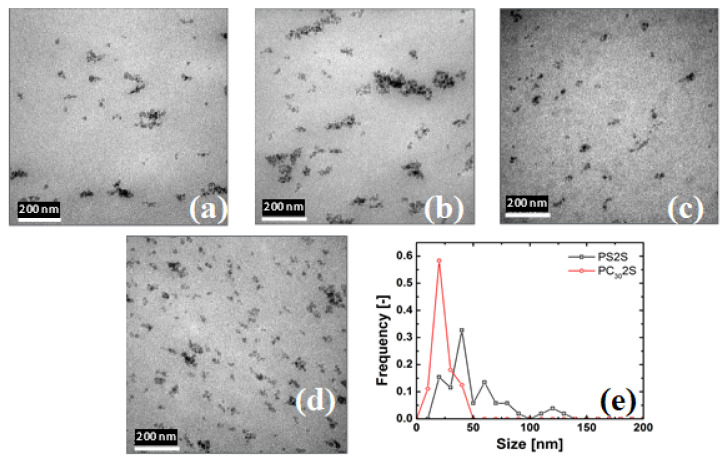
TEM micrographs of the nanosilica composite: (**a**) PS with 0.5 vol% nanosilica, (**b**) PS with 2.0 vol% nanosilica, (**c**) PC30 with 0.5 vol% nanosilica (**d**) PC30 with 2.0 vol% nanosilica, and (**e**) particle size and distribution in PS and PC nanocomposites. The primary particle of nanosilica is 12 nm and the scale bar in all the figures are 200 nm.

Figure 1 shows the dispersion of nanosilica in PC30 and PS matrices as a function of its concentration. The TEM micrographs of the PS/silica composites depicted in Figure 1a,b show that the silica particles form agglomerates with an average diameter of 80–200 nm. At a concentration of 0.5 vol% nanosilica, agglomerates sizes range from 80–100 nm along with a few primary nanosilica particles, while at 2.0 vol% nanosilica, the dispersion quality deteriorates with aggregate sizes increasing up to 200 nm. In contrast, Figure 1c,d for PC-30/silica nanocomposites show a better dispersion. The agglomerate sizes are smaller with an average diameter of 25–50 nm with more primary particles visible; however similar to the PS/silica systems increasing the nanosilica concentration leads to larger agglomerates. The general improvement of dispersion can be attributed to the interaction between the polar silica surface and the polar PC compared to the apolar PS, see Figure 1e. As mentioned in the experimental section the size distribution was calculated using 20 pictures taken from different parts of the sample. The TEM image shown in the figure below closely follows the size distribution and therefore can be taken as representing the sample.

### 4.2. Evidence of Grafting

**Figure 2:**
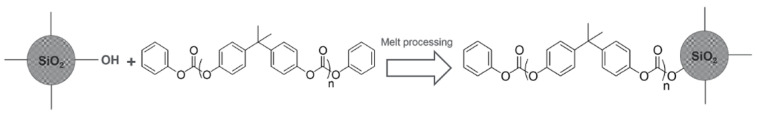
Scheme and possible molecular structure formed during the reaction between PC and the nanosilica particle during melt-compounding.

It is hypothesized that during the melt compounding of the PC and pure ${\mathrm{SiO}}_{2}$, a reaction of the carbonate group with the surface hydroxyl groups of the pure ${\mathrm{SiO}}_{2}$ occurs as shown in Figure 2.

**Figure 3:**
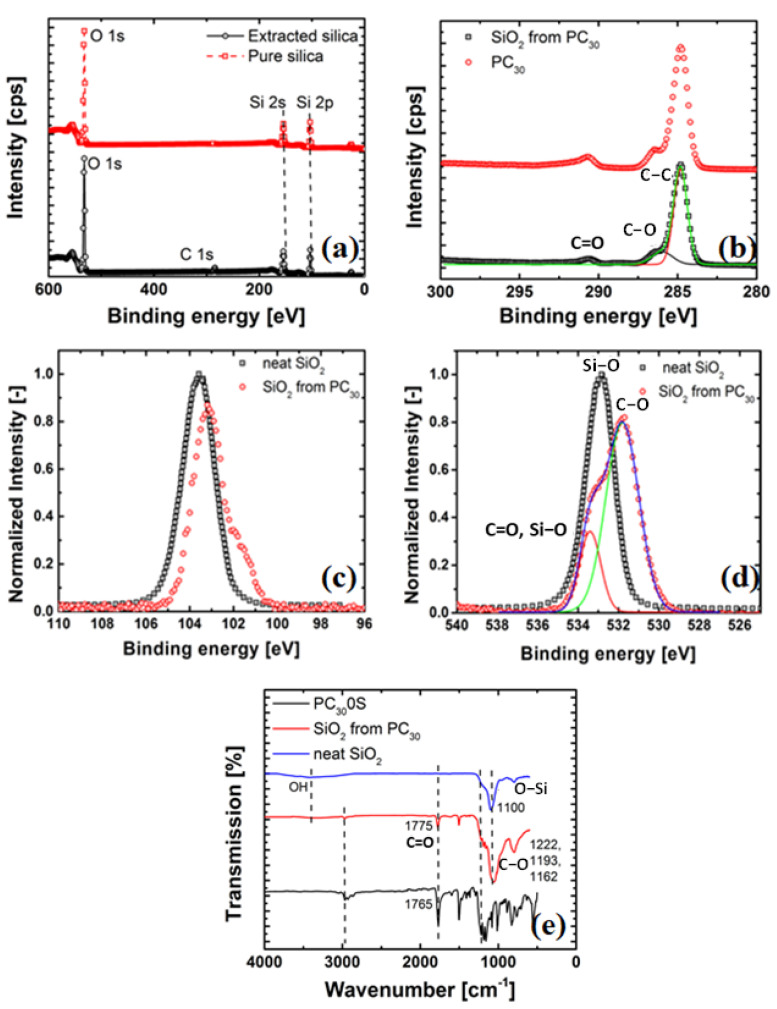
Spectra of: (**a**) pure ${\mathrm{SiO}}_{2}$ compared with extracted-${\mathrm{SiO}}_{2}$ and (**b**) peak-fitting results of C1s, (**c**) Si2p of pure and extracted silica from ${\mathrm{PC}}_{30}$, (**d**) O1s of pure and extracted silica from ${\mathrm{PC}}_{30}$ Compliance of the ${\mathrm{PC}}_{30}$ and ${\mathrm{PC}}_{30}$/silica (0.7 vol%) nanocomposites, and (**e**) FTIR spectra of extracted ${\mathrm{SiO}}_{2}$ compared with pure ${\mathrm{SiO}}_{2}$ and PC. The curves are vertically shifted to make a comparison between extracted and pure nanosilica and PC.

To verify whether grafting of the polymer chain takes place during melt extrusion, FTIR and XPS spectroscopy were used. Since the grafting reaction of PC chain segments only occurs at the surface of the silica, using these techniques on the nanocomposite (bulk) samples might not give enough evidence for the grafting reaction, see Figure 3.

Figure 3a shows the XPS spectra and the results of the C1s from the extracted (from PC) and pure ${\mathrm{SiO}}_{2}$. It can be observed that in the extracted ${\mathrm{SiO}}_{2}$ from the ${\mathrm{PC}}_{30}$, a new peak at the binding energy of 285.00 eV is seen. This peak can be attributed to the C1s from the PC based on the peak fitting of the C1s (see, Figure 3b). There are three peaks at around 285.00 eV, i.e., peak at the binding energy 284.70 eV assigned to C–C group, a peak at 286.00 eV corresponding to the C–O group and s peak at 291.00 eV assigned to C=O group. Furthermore, the XPS spectrum of the Si2p in Figure 3c shows that the Si2p peak of extracted ${\mathrm{SiO}}_{2}$ at 103.20 eV is lower than that of pure ${\mathrm{SiO}}_{2}$ (103.80 eV) and this chemical shift can be ascribed to the formation of Si–O–C bonds (i.e., the nanoparticle bonded to PC). This new bond slightly shifts the binding energy. Finally, in Figure 3d, the peak fitting results for the O1s peak are shown. The O1s peak of extracted ${\mathrm{SiO}}_{2}$ at 532.55 eV assigned to Si–O bonds is lower than that of pure ${\mathrm{SiO}}_{2}$ (533.55 eV). As the carbonyl groups (–C=O–) assumed to be bonded with Si–O bonds are electron withdrawing groups, the conjoint oxygen atoms on Si–O bonds become electron deficient resulting in a shift of the peak of O1s towards the high binding energy direction.

These results are further supported by FTIR spectroscopy, as previously discussed in [46]. It would suffice to mention here that FTIR spectra shown in Figure 3e reveals that the carbonyl group of the PC chains is bound to the ${\mathrm{SiO}}_{2}$ particles, which results in the downshift of the C=O stretching. All observations above strongly indicate that the ${\mathrm{SiO}}_{2}$ surface is grafted with PC as a result of its reaction with the surface hydroxyl groups present in the ${\mathrm{SiO}}_{2}$ as depicted in Figure 2. Finally, the nanosilica extracted from PS showed no difference in the binding energy shift or appearance of FTIR peaks (not shown).

We would like to note here that the lengths and density of the grafts could not be determined exactly as the reaction between the nanosilica and the polydisperse polymer matrix is random. It is postulated that the lengths of the grafts follow a polydisperse distribution with the average length in the order of the molar mass between entanglements ($M_{e}$) of PC, and the density to be sparse. Furthermore, it was difficult to estimate the exact number of terminal hydroxyl groups on the silica surface and thus difficult to estimate the number of PC chains that might have reacted. However, as an estimate, the ratio of the intensity of hydroxyl peaks might be used. Using this approach, we can say that 35–40% of the OH on the surface have reacted.

**Figure 4:**
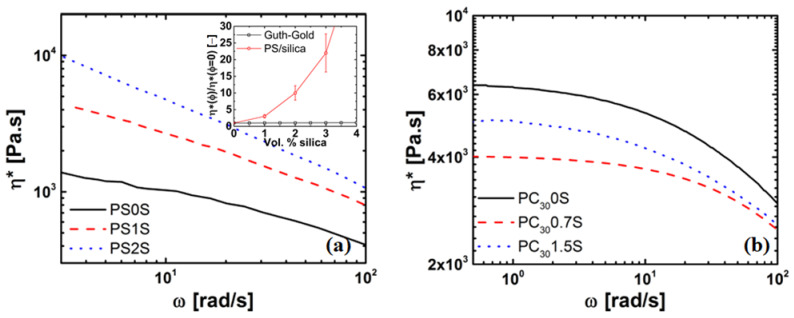
Log-log plot of the complex viscosity $\eta^{*}$ versus angular frequency (ω) for: (**a**) PS/silica nanocomposites and inset viscosity ratio (at 1 rad/s) & Guth–Gold predictions and (**b**) PC/silica nanocomposites at *T*–$T_{g}$ of 100 ° C.

**Figure 5:**
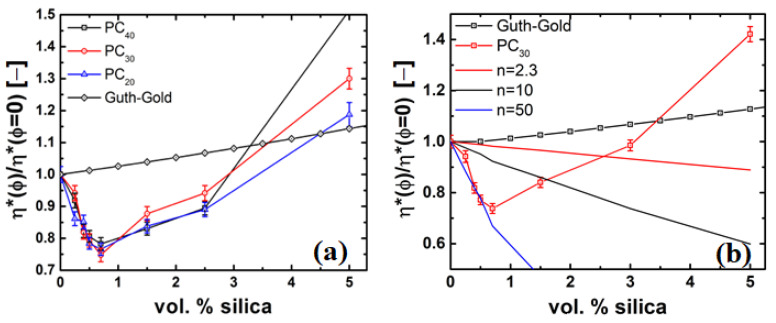
Viscosity ratio and Guth–Gold predictions as a function of filler loading in: (**a**) ${\mathrm{PC}}_{30}$/silica nanocomposites with different molar masses of PC uncorrected for molar mass, and (**b**) effect of plasticization on viscosity as a function of nanosilica particle concentration.

On the addition of nanosilica particles, the PS/silica composites show an increase in viscosity over the whole frequency range, and do not reach a Newtonian plateau. The prediction of the Guth–Gold and viscosity ratio of the PS systems is shown in the inset. It can be seen that the viscosity increase is much stronger than predicted by the Guth–Gold relation, suggesting that the particle agglomeration, chain bridging (i.e., where the fillers are linked by the polymer chains in between them) and formation of a particle network, and therefore hydrodynamically reinforces the PS matrix [58]. In contrast, the PC/silica composites reach a Newtonian plateau with the viscosity decreasing at low concentration and increasing at higher concentrations, i.e., ϕ > 0.005 or 0.5 vol%, compared to unfilled PC, see Figure 4b. For ϕ > 0.7 vol%, the Newtonian plateau shifts to lower frequencies for the PC/silica nanocomposites as compared to unfilled PC, but both unfilled and PC nanocomposites show similar slopes at high frequencies. This is further exemplified in Figure 5, where the ratio of the complex viscosity (at 1 rad/s) of the nanocomposite to the unfilled polymer is plotted, i.e.,

(11) $$\left|\frac{\eta^{*}\left(\phi \right)}{\eta^{*}\left(\phi =0\right)}\right|vs.\;\omega$$

We would like to note here that we focused on the terminal viscosity behaviour, as the effect of viscosity drop was most significant.

### 4.3. Rheology of Silica Nanocomposites

The rheological properties of the polymer nanocomposite are affected by the dispersion of nanoparticles. Figure 4 shows a plot of the complex viscosity, $\eta^{*}$, as a function of frequency, ω, at a temperature of *T*–$T_{g}$ = 100 °C for the PS/silica and PC/silica nanocomposites. As can be seen in Figure 4a, unfilled PS reaches a Newtonian plateau at low frequencies (<1 rad/s). Figure 5a can be basically divided into two parts, i.e., a decrease in viscosity of 25% (for ${\mathrm{PC}}_{30}$/silica nanocomposite) from 0–0.7 vol% of nanosilica and is independent of molar mass, and a region of >0.7 vol%, where the viscosity increases exponentially and is dependent on molar mass. The former phenomenon is not intuitive hinting to strong polymer-particle interaction. The latter is attributed to particle-particle interaction resulting in agglomeration and formation of particle networks. At low frequencies, the relaxation time of the of these networks are large and thus dominates the viscosity of the nanocomposites.

**Figure 6:**
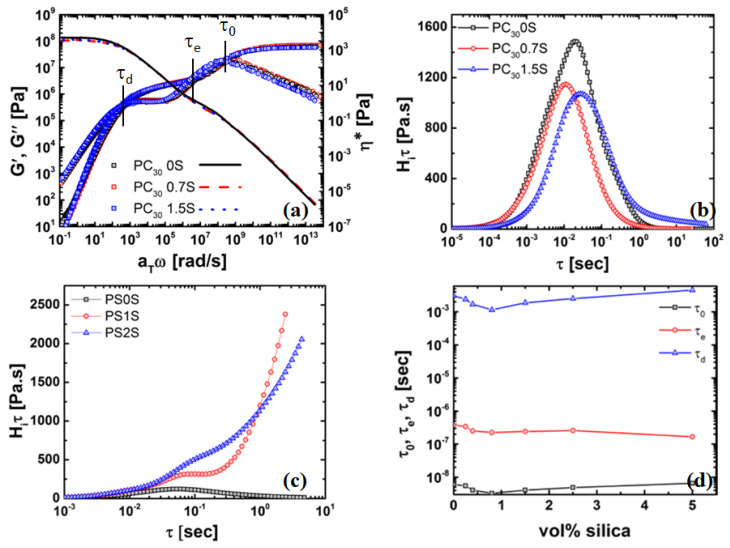
(**a**)Mastercurve for PC/nanocomposite, continuous relaxation spectra from the mastercurve for (**b**) ${\mathrm{PC}}_{30}$ and ${\mathrm{PC}}_{30}$/silica nanocomposite and (**c**) PS/silica nanocomposites, and (**d**) the characteristic time scales, i.e., the monomer relaxation ($\tau_{0}$), the relaxation time of one entanglement segment ($\tau_{e}$), and the reptation time ($\tau_{d}$) are accelerated are ϕ≤0.7%.

It can also be seen that the upswing of viscosity is dependent on the molar mass of the PC matrix. This dependence stems from the fact that the terminal shear viscosity is proportional the molar mass. The increase in the case of ${\mathrm{PC}}_{40}$ is primarily due to the higher dispersity in the $M_{z}$ region. These behaviours are further elucidated in Figure 6b,c, where the relaxation spectra of the PC and PS nanocomposites are shown. The above observations clearly suggest that both polymer-particle and particle-particle interactions are important and play a major role in the observed viscosity behavior.

Kim et al. [59] showed that grafted nanoparticles could act as plastizizers, in order to check if this is the case in our case the data is fit to the following relation.

(12) $$\frac{\eta }{\eta_{0}}={\left(1-\phi \right)}^{n}$$

where *n* > 0 and denotes the extent of plasticization, in most cases 2 < *n* < 2.3 [2,26,60].

Common value of *n* based on the scaling laws (i.e., contour length per unit volume; increasing plasticizer decreases the length per unit volume) is 2, experimental results indicate n 2.3, for neutral plasticizers [61]. Figure 5b shows the failure of Equation (12) with *n* = 2.3 and 10 describe the decrease in viscosity. While the data can be fit with *n* = 50 for ϕ ≤ 0.7 vol%, it must be realized the description is not appropriate. A very high value of *n* indicates a dramatic decrease in contour length per volume over a very small concentration of the plasticizer, which is physically not feasible. However, as noted by [62], values of *n* > 2.3 could mean strong interactions between the polymer and plasticizer. Therefore, the viscosity drop cannot be entirely attributed the plasticizing effect of nanosilica, and such large values of *n* indicate that there are other governing physics playing a role.

Figure 6 shows the master curve for PC/silica nanocomposites, along with their respective relaxation spectrum. TTS was performed with only a horizontal shift (shift factors are given in supplementary information Figures S4 and S5). This suggests that the viscosity behavior is primarily related to the nanoparticles affecting polymer dynamics rather than particle dynamics. For PC nanocomposites, (Figure 6a), all mastercurves showed terminal slopes of 1 and 2 for $G^{\prime }(\omega$) and $G^{\prime \prime }(\omega$), respectively. The plateau modulus ($G_{N0}$) was in the order of 2.0 × $10^{6}$ Pa for all cases and in-line with literature [44,63,64]. We would like to point out here that plasticization would have resulted in a decrease of $G_{N0}$ [59], thus supporting the above claim that plasticization is not the likely cause.

The observations in Figure 6 are further supported by the weighted-average relaxation time spectra (Equations (2) and (3)) and shown in Figure 6b–d. It is clear that peak relaxation and other characteristic time scales for the PC-based nanocomposites follows the same trend as the viscosity drop at 1 rad/s, i.e., relaxation time (0.7 vol%) < relaxation time (0 vol%) < relaxation time time (1.5 vol%), which is in contrast to the PS nanocomposites, where there is a continuous increase in viscosity (and therefore peak relaxation time) with increasing the nanoparticle concentration. Both PC nanocomposites with 1.5 vol% nanosilica and all the PS/nanocomposites shows a tailing and upswing in the relaxation time spectrum (indicating very long relaxation times), which supports the above claim that this regime is controlled by strong particle-particle interactions, i.e., nanosilica network due to agglomerating particles, along with chain-bridging. Similar observations were made by other research groups in PS/silica, PEO/silica nanocomposites [58,65,66]. Various hypotheses to explain the terminal viscosity drop and associated the acceleration of time scales will be discussed in the sections below.

### 4.4. Possible Mechanisms of Viscosity Reduction

**Figure 7:**
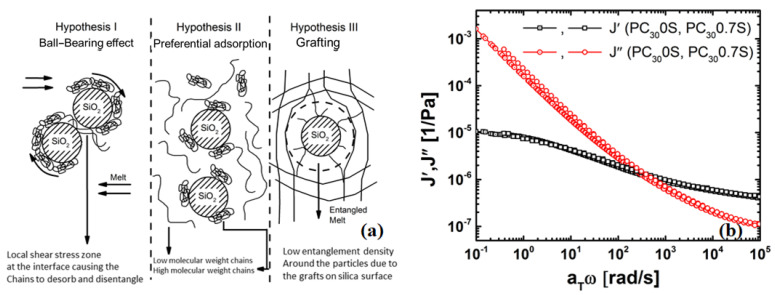
(**a**) Hypotheses for the observed viscosity drop in PC/silica nanocomposites., and (**b**) Compliance of the ${\mathrm{PC}}_{30}$ and ${\mathrm{PC}}_{30}$/silica (0.7 vol%) nanocomposites. At $J^{\prime }\left(\omega =0\right)$, the low frequency limiting value of $J_{0}$ is the steady-state compliance.

The following hypotheses were considered for the mechanism causing the viscosity reduction due to the grafted particles (Figure 7):

The ball-bearing mechanism [21,67,68], see Figure 7a (left), cannot occur as low shear rates (or frequencies) as the grafting and high melt viscosity of the matrix hinders the rotation of the particles under shear flow. The selective physisorption of polymer chains on the nanoparticle surface postulated [18] (Figure 5a) (middle) is also not plausible as any adsorption is hindered by the grafting. Furthermore, no changes in the steady state compliance ($J_{0}$) were observed for PC and its nanocomposite [49,69], see Figure 5b indicating that there is no selective adsorption. The changes in $J^{\prime \prime }\left(\omega \right)$ however indicate that the viscous loss can be attributed PC-grafted silica interactions.

#### 4.4.1. Chain Grafting Causing Disentanglement at the Interface Enabling Faster Modes of Relaxation

Though studies have shown an increase in entanglement density and friction [70], our findings, discussed below, indicate that the sparsely grafted nanosilica could result in a decrease in entanglement density around the nanoparticle resulting in a Rouse-like zone, where the number of entanglements is far less than the bulk. And beyond a critical concentration of nanosilica, bridging of chain segments between particles and particle agglomeration is expected to take over, resulting in an increase of viscosity [6,71,72].

#### 4.4.2. Thickness of the Grafted Layer of the Polymer Chain around the Particle

**Figure 8:**
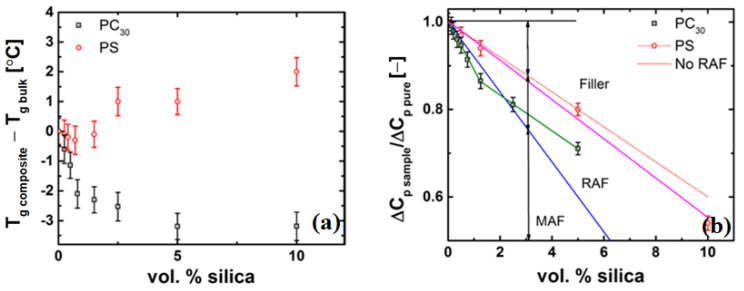
(**a**) in $T_{g}$ as a function of nanosilica concentration for PC and PS silica nanocomposites, and (**b**) heat capacity ratio of the nanocomposite with respect to the unfilled polymer as a function of silica concentration for PC and PS silica nanocomposites.

Figure 8 shows the difference in $T_{g}$ of the homopolymer and the respective PS and PC nanocomposites. The PC/silica nanocomposites show a decrease in $T_{g}$ up to 3 °C, while the PS nanocomposites show a slight increase. This result, though counter intuitive, i.e., favourable interactions increase the $T_{g}$ and vice versa, can be explained. For the case of PS, hydrophobic PS and hydrophilic silica have a large surface energy difference and thus are non-wetting to each other (i.e., the PS and the silica particles to remain ‘well-separated’ with higher mobility of polymer chains at the interface of the nanoparticle). However, it is also likely that there is some immobilization of the polymer chains on the surface resulting in large relaxation times and viscosity upswing as seen and described in above section. Similar behaviour has been observed in other studies [58,65,73] that include PS/Silica nanocomposties.

In the case of PC-based systems, the decrease in $T_{g}$ decrease could be explained by changes by the increased mobility of the chains at surface that allow for availability of faster relaxation modes, this is also evident from the relaxation spectrum discussed above Figure 6b. This could be possible if there is a Rouse-like zone around the particle. Similar behaviour was also seen by [74] for bi-disperse PS grafted nanosilica dispersed in PS.

**Figure 9:**
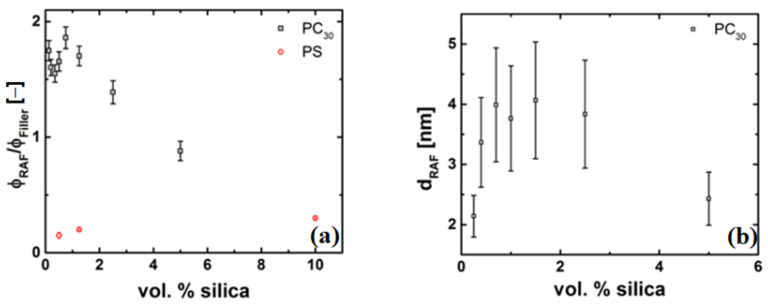
(**a**) Normalized RAF for PC and PS silica nanocomposites with the different regimes and, (**b**) Thickness of the RAF layer around the silica nanoparticles.

The RAF is used as a measure of the thickness of the grafted layer around the nanoparticle. The RAF is shown in Figure 9 for both the PC and PS/silica nanocomposites. The dashed line (red) represents the case when no RAF, i.e., RAF = 0, is present and is plotted from Equation (10). The data for the PS nanocomposite follows the no RAF line closely, suggesting that there is no grafted or much of an immobilized layer of polymer on the nanoparticle. However, in the case of the PC nanocomposites, the decrease in the ratio of heat capacity is much steeper than the RAF = 0 line, and shows two slopes, i.e., one slope up to a concentration of approximately 1.5 vol% and another slope beyond a concentration of 1.5 vol%. For ϕ > 1.5 vol% the slope of the line decreases and tends more towards the RAF = 0 line. This change in slope can be a result of agglomeration of the silica particles, which decreases the surface area, thereby reducing the amount of polymer chains grafted on the particle surface.

Furthermore, Equation (9) assumes a constant RAF around all particles. This assumption is not well justified by the data in Figure 8b for the case of the PC nanocomposite as shown in Figure 9a. If all the nanoparticles were covered by the same amount of polymer chains, one would expect to find a constant ratio between the RAF and the filler content. The decrease of the ratio indicates that a decrease of the RAF per nanoparticle with increasing filler concentration occurs, which might be due to agglomeration of the nanoparticles. Since our focus lies at low concentrations, we do not follow the correction as mentioned in Equation (8). The grafting of the polymer chains should also affect the enthalpy relaxation below the glass transition [42,75]. With the evidence that RAF is present in the case of the PC nanocomposites, the thickness of this layer needs to be determined, as this is crucial for explaining the observed viscosity drop. Using a diameter of 12 nm and a density of 2.4 g/${\mathrm{cm}}^{3}$ for the ${\mathrm{SiO}}_{2}$ filler, a layer thickness ranging from 3 to 5 nm was calculated using the approach mentioned by Schick and Donth [76]. The thickness of the RAF as a function of silica concentration is plotted in Figure 9b.

#### 4.4.3. Evidence of a Low-Viscosity Layer

**Figure 10:**
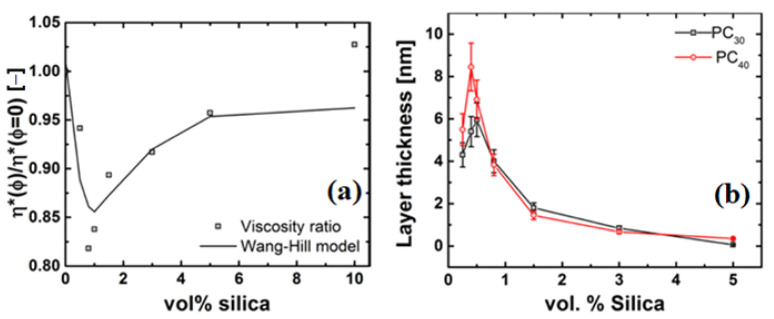
(**a**) Wang–Hill model fit to viscosity ratio in ${\mathrm{PC}}_{30}$ and, (**b**) low-viscosity layer thickness predicted by the Wang–Hill model.

TM-DSC experiments show that the thickness of the grafted layer (l) on the nanosilica is of the order $l_{grafted\;layer}$ ≤ $R_{e}$ < $r_{particle}$. Kalfus et al., Jiang et al., and Picu and Rakshit showed that the surface interaction is not confined to a surface-bound layer, and can have a far-field effect on the order of at least $R_{g}$ [33,36,77]. The influence zone of the grafted layer can be examined using concepts recently published by Ganesan and Pryamitsyn, and Wang and Hill [78,79]. As the Ganesan-Pryamitsyn model focused on unentangled polymer nanocomposites, we therefore use the Wang-Hill model. The Wang-Hill model is a continuum model that captures the terminal shear viscosity behavior of nanocomposites (see Supplementary Material for description, Section 3). The model uses the concept of a layer at the nanoparticle-polymer interface which has a different density and viscosity from the bulk and has been successful in describing the non-Einstein like viscosity behavior reported by Mackay and co-workers [12,15,16]. The existence of such a layer with a different density (ρ) and viscosity (η) was also found in various numerical simulations [3,80,81,82,83,84]. Using a critical molar mass ($M_{c}$) of 5000 g/mol for PC24 the viscous layer (δ) for PC/silica nanocomposite at 250 °C is calculated and given in Table 2 and shown in Figure 10. The values of [η] are calculated using Equation (1) with the viscosity values obtained from the rheology experiments. It is important to note that the model accounts for only bare particle-polymer interactions, but can give qualitative predictions of the phenomena, validate the physics and hypothesis of the presence of a low viscosity layer around the particle.

This model is able to qualitatively predict the viscosity drop. As the model does not account for particle–particle interaction, it does not describe the viscosity increase. Thus, for the PC/silica nanocomposite samples, the layer thickness strongly depends on the actual filler degree and for the smallest silica fractions δ (0.7 vol%) = 7 nm and δ (3.0 vol%) = 1 nm.

The results indicate that the influence zone in order to achieve the observed viscosity drop is approximately the $R_{g}$, irrespective of the molar mass of PC. Therefore, around the particle at a distance of $R_{g}$, a low viscosity layer leads to an overall drop in viscosity. This lower viscosity layer most likely is present due to a reduced number of entanglements around the particle, as a result of constraint release-like mechanism at the nanoparticle-polymer interface occurring due to the grafts. This observation is consistent with the results found by earlier work [14,15,19].

As shown by [85,86], for $r_{particle}$ < $R_{e}$ and times $\tau_{e}$ < t < $\tau_{d}$, the particle experiences Rouse dynamics of the matrix polymer chains, i.e., the nanoparticle motion is influenced by the rearrangements of the surrounding polymer chain segments. This could change the entanglement density resulting in a lower bulk viscosity by allowing the nanoparticles to diffuse very fast. At times t > $\tau_{d}$ the motion of the particles is diffusive. For particles $R_{e}$ < $r_{particle}$ < $R_{g}$, at $\tau_{e}$ > t the particles experience Rouse dynamics of the matrix chains and at $\tau_{e}$ < t, the particle motion is constrained by entanglements and in order to move, they have to wait for the polymer to relax by reptation and thus do not contribute to reduction in the viscosity of the melt. However, if the particles are slightly larger than the tube diameter, i.e., as in this case where the $R_{e}$ = 4 nm and the $r_{particle}$ = 9 nm, they do not have to wait for the whole polymer to relax and can diffuse by hopping between entanglements. This means that at longer times (towards terminal relaxation), the motion of the nanoparticles results in the grafts on the surface participate in reducing the number of entanglements in the surrounding of the particle, i.e., constraint release-like mechanism, thus resulting in a low viscosity layer at the nanoparticle-polymer interface of the order of $R_{g}$. This claim is further supported by the fact that the relaxation times, i.e., $\tau_{d}$, $\tau_{e}$, $\tau_{0}$, are faster at low concentrations as shown earlier. Furthermore, as shown by Mangal et al. [19] insights on how the polymer chains relax in the grafted nanosilica environment can be obtained by taking the ratio of the characteristic time scales, i.e.

$\kappa =\left(\tau_{e}/\tau_{0}\right)(1/4)=R_{e}/b$, (short time scales), where *b* is the Kuhn length.
$\epsilon =\left(\tau_{d}/\tau_{e}\right)=3Z^{3}$ (long times scales)

**Figure 11:**
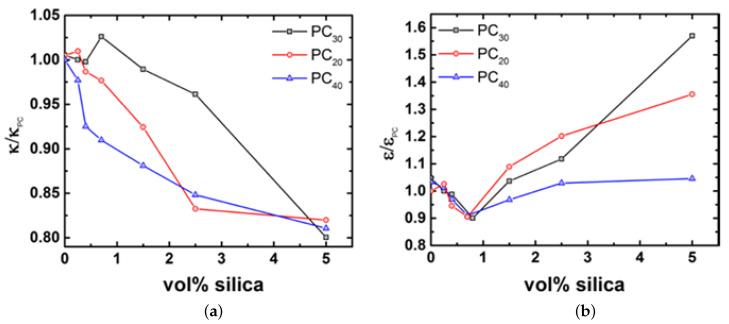
(**a**) Relative tube diameter and (**b**) relative number of entanglements for PC and PC/silica nanocomposites normalized with respect to unfilled PC.

Figure 11a shows that irrespective of the sample, κ remains unchanged until a concentration of 0.7 vol% nanosilica. Beyond this concentration, κ decreases. This implies that at shorter times and larger concentrations of nanosilica particles (>0.7 vol%) the PC effectively has a smaller tube diameter. This implies that the nanoparticles exert additional constraints on the tube. Correspondingly, Figure 11b shows that for longer time scales, irrespective of the molar mass of the PC, there is a decrease in the number of entanglement or ϵ at low concentrations (up to 0.7 vol%) followed by an increase at concentrations of nanosilica. Therefore the physics of lowering the viscosity and formation of the disentangled layer is a competition between the diffusing nanosilica particles destroying entanglements and the relaxation on the bulk PC chains by reptation. These processes occur at a similar time scale thereby enabling constraint release [19]. These conclusions are also consistent with predictions from simulations and theory [87,88,89,90,91] and explains the earlier onset of reptation of the polymer chains as observed earlier in the continuous relaxation spectrum.

## 5. Conclusions

A series of polycarbonate and polystyrene silica nanocomposites prepared by melt extrusion were experimentally analyzed to provide fundamental insights on the non-Einstein-like viscosity behaviour. Under small amplitude oscillatory shear experiments conditions, the linear rheological properties of each nanocomposites were found to be different from the corresponding properties in its matrix at the terminal regime. The viscosity of the PC nanocomposite was found to decrease at ≤ 0.7 vol% of nanoparticles and increase thereafter. The PS nanocomposite on the contrary only showed an increase. In both cases the increase was found to be much larger than as predicted by the Einstein relation, and was attributed to agglomeration and chain bridging. FTIR, XPS and TM-DSC analysis showed strong evidence polycarbonate chains grafted to nanosilica particles. Analysis of the DSC and rheology experiments suggests that the thickness of the grafted PC layer on the silica surface is larger than the cooperatively rearranging regions, and was the order of the tube diameter ($R_{e}$), i.e., 2–4 nm, suggesting that the grafts must be of the order of ≥$M_{e}$ and most likely polydisperse. The grafting was also found to speed up the chain dynamics, resulting in a decrease in *T_g_* and speeding up of the relaxation, i.e., $\tau_{d}$, $\tau_{e}$, and $\tau_{0}$, at ≤0.7 vol% silica nanoparticles. At short time scales and high concentrations (>0.7 vol%), the nanoparticles imposed additional constraint on the tube and result in an earlier onset of reptation relaxation. At longer times scales and low concentrations, i.e., <0.7 vol%, the grafts appear to reduce the number of entanglements of the host and accelerate tube escape via constraint release. Finally, the Wang–Hill model qualitatively shows the presence of a low viscosity layer of the order of $R_{g}$ of the PC chain around the particle. Finally, the observed effect is not dependent on the kind of PC, as it depends only on the nature of the reactive groups.

## Figures and Tables

**Table 1:** Overview of the material characteristics. $R_{g}$ and $R_{e}$ are obtained from [44].

Material	$M_{w}$ [Kg/mol]	PDI	$R_{g}$ [nm]	$R_{e}$ [nm]	$T_{g}$ [°C]
${\mathrm{PC}}_{20}$	21.3	2.3	6.4	3.8	142
${\mathrm{PC}}_{30}$	31.5	2.5	7.6	3.8	148
${\mathrm{PC}}_{40}$	39.3	3.2	8.5	3.8	149
PS	300	2.6	14.5	8.4	100

**Table 2:** Low viscosity layer thickness calculated using the Wang–Hill model for PC/silica nanocomposites.

Material	[η]	Layer Thickness [nm]
${\mathrm{PC}}_{20}$0S, ${\mathrm{PC}}_{30}$0S, ${\mathrm{PC}}_{40}$0S	0	-
${\mathrm{PC}}_{20}$0.7S	−13	6
${\mathrm{PC}}_{20}$3S	−1.4	1
${\mathrm{PC}}_{30}$0.7S	−13	7
${\mathrm{PC}}_{30}$3S	−1.4	0.8
${\mathrm{PC}}_{40}$0.7S	−16	8
${\mathrm{PC}}_{40}$3S	−2.7	0.9

## Data Availability

Data are contained within the article.

## Supplementary Materials

The following are available online at The following are available online at https://www.mdpi.com/article/10.3390/nano11071839/s1, Figure S1: Corrected viscosity drop using molar mass for ${\mathrm{PC}}_{30}$. There were no changes seen in case of PS., Figure S2: Parallel plate and capillary rheology data for (a) ${\mathrm{PC}}_{30}$, (b) ${\mathrm{PC}}_{30}$0.7S at 280 °C, Figure S3: Parallel plate and capillary rheology data for PS: (a) PS0S, (b) PS2S at 200 °C, Figure S4: Shift factors employed in obtaining TTS master curves for PC: (a) ${\mathrm{PC}}_{30}$, (b) ${\mathrm{PC}}_{30}$0.7S, and (c) ${\mathrm{PC}}_{30}$1.5S. The solid line is the WLF equation fit, Figure S5: Shift factors employed in obtaining TTS master curves for PS: (a) PS0S, (b) PS1S, and (c) PS2S. The solid line is the WLF equation fit., Table S1: Molar masses after compounding and measurements., Table S2: Corrected viscosity drop incorporating the change in molar mass.
