# Supplementary material for: Anomalous Terminal Shear Viscosity Behavior of Polycarbonate Nanocomposites Containing Grafted Nanosilica Particles
Source: Nanomaterials (Basel). 2021 Jul 15;11(7):1839. doi: 10.3390/nano11071839 (PMC8308399; doi:10.3390/nano11071839)
Supplement: Supplementary file 1 [file nanomaterials-11-01839-s001.zip › nanomaterials-1267887-supplementary.pdf]

nanomaterials

Article

Anomalous Terminal Shear Viscosity Behavior of Polycarbonate Nanocomposites Containing Grafted Nanosilica Particles

Vaidyanath Ramakrishnan [1,*,†], Johannes G. P. Goossens [2,*,†] and Theodorus L. Hoeks [2,†] and Gerrit W. M. Peters [1]

[1] Department of Mechanical Engineering, Eindhoven University of Technology, P.O. Box 513, 5600 MB Eindhoven, The Netherlands; g.w.m.peters@tue.nl
[2] Department of Chemical Engineering and Chemistry, Eindhoven University of Technology, P.O. Box 513, 5600 MB Eindhoven, The Netherlands; theo.hoeks@sabic.com
* Correspondence: Correspondence: vaidyanath.ramakrishnan@sabic.com (V.R.); han.goossens@sabic.com (J.G.P.G.)
† Current address: SABIC, Plasticslaan 1, 4612 PX Bergen op Zoom, The Netherlands.

Citation: Ramakrishnan, V.; Goossens, J.G.P.; Hoeks, T.L.; Peters, G.W.M. Anomalous Terminal Shear Viscosity Behavior of Polycarbonate Nanocomposites Containing Grafted Nanosilica Particle. Nanomaterials **2021**, 11, 183. https://doi.org/10.3390/nano11071839

Academic Editor(s): Carola Esposito Corcione

Received: 4 June 2021
Accepted: 9 July 2021
Published: date

Publisher's Note: MDPI stays neutral with regard to jurisdictional claims in published maps and institutional affiliations.

Copyright: © 2021 by the authors. Submitted for possible open access publication under the terms and conditions of the Creative Commons Attribution (CC BY) license (http://creativecommons.org/licenses/by/4.0/).

1. Effect of Wall Slip In-homogeneous Flow and Molar Mass Decrease.

The following two potential unwanted side effects that could be responsible for the viscosity drop are discussed:

1.1. Slip or In-homogeneous Flow

1.2. Molar Mass Degradation during Fabrication and Measurements.

Henson et al. showed that a decrease in viscosity of nanocomposites could be due to in-homogeneous flow or slip at the slit wall caused by the nanoparticles [1] As all rheological measurements reported were performed by using a parallel-plate geometry, the possible occurrence of wall slip can be determined by performing measurements for different gaps. No significant effect was observed when the gap distance was varied from 0.5 to 1.5 mm for both PC and PS nanocomposites. This indicates that the decrease in viscosity does not originate from wall slip or in-homogeneous flow.

The second possibility is the degradation of the polymer nanocomposite during the preparation or measurement. It is known from the molar mass dependence of the zero shear viscosity, $\eta = kM_w{}^{3.4}$ that a slight decrease in molar mass can lead to a significant decrease in viscosity. To ensure that the decrease in viscosity is not due to the degradation of the PC or PS, the molar masses of all nanocomposites after the preparation were characterized by SEC. The results are summarized in Table S1, and it can be observed that a small decrease in the molar mass and a corresponding small increase of the polydispersity index (PDI) occur during the extrusion process after the addition of silica.

Table S1. Molar masses after compounding and measurements.

Material	M_w ± 0.5 (after compounding)	PDI (after compounding)	M_w ± 0.5 (after rheology)	PDI (after rheology)	Change in M_w	Change in M_w (due to silica, after rheology)
	[kg/mol]		[kg/mol]		[kg/mol]	[kg/mol]
**PC$_{20}$0S**	21	2.33	21.2	2.33	0.2	
**PC$_{20}$0.7S**	20.7	2.34	20.8	2.34	0.1	−0.3
**PC$_{20}$1.5S**	20.6	2.36	20.4	2.36	−0.2	−0.8
**PC$_{30}$0S**	30.2	2.55	30.1	2.54	−0.1	
**PC$_{30}$0.7S**	30.1	2.55	30.6	2.54	0.4	−0.5
**PC$_{30}$1.5S**	30.2	2.57	29	2.57	−0.8	−0.9
**PC$_{40}$OS**	40	3.23	39.8	3.21	−0.2	
**PC$_{40}$077S**	39.3	3.26	38.9	3.25	−0.4	−0.9
**PC$_{40}$1.5S**	39	3.23	38.7	3.23	−300	−1100
**PS0S**	300	2.6	300	2.61	0	
**PS1S**	300	2.62	300	2.61	0	0

After correcting the viscosity for the change in molar mass using the power law equation, the remaining decrease in the viscosity for the system with the highest viscosity reduction, i.e. ~ 0.7 vol% silica, accounts to ~ 20 %, as shown in Figure S1 and Table S2. To exclude possible thermo-oxidative degradation reactions, the molar mass of the samples before and after the rheological measurements was also measured and the molar mass variations observed were negligible. Therefore, thermal degradation hardly occurs during rheology measurements.

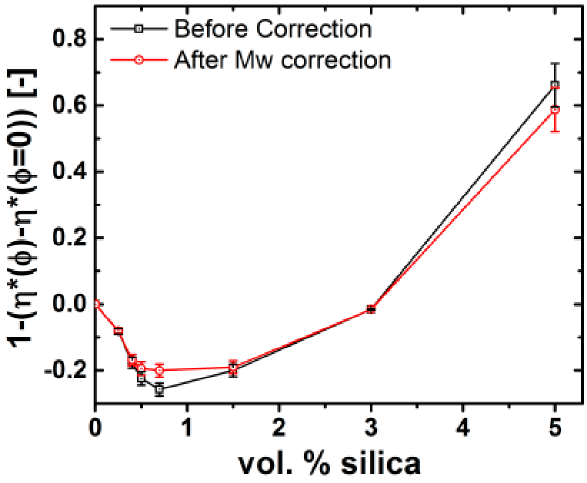

Figure S1. Corrected viscosity drop using molar mass for PC_{30}. There were no changes seen in case of PS.

Table S2. Corrected viscosity drop incorporating the change in molar mass.

Material	Δ before correction [%] ± 3%	Δ after correction [%] ± 3%
$PC_{20/30/40}$ PS0S	0	0
PC_{20}0.7S	−26	−20
PC_{30}0.7S	−25	−20
PC_{40}0.7S	−26	−19
PS1S	400	400

2. Cox-Merz rule:

As mentioned in the article, the systems don't follow the Cox-Merz rule, i.e. f(w)=f($\dot{\gamma}$), where w is the frequency as measured in a parallel-plate measurements and $\dot{\gamma}$ is the shear rate from capillary rheology. The graphs below for PC and PS systems clearly show that the data obtained via capillary rheology and parallel-plate rheometry cannot be superimposed. We would like to note here that the capillary rheology measurements were performed a Göttfert Rheograph 2000 machine at temperatures of 280 °C and 200 °C for PC and PS respectively in a shear rate range between100 and 3000 s^{-1}. A L/D ratio of 30 was used and the capillary diameter used was 1mm. For the capillary experiments, the true wall shear rate has been calculated by using the Weissenberg-Rabinowitch correction.

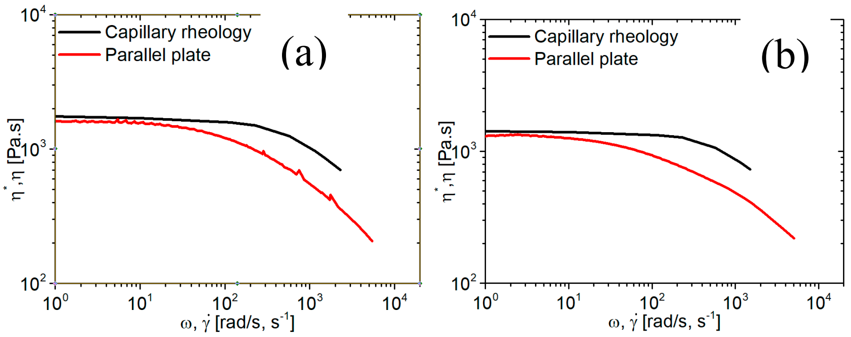

Figure S2. Parallel plate and capillary rheology data for (**a**) PC30, (**b**) PC300.7S at 280 °C

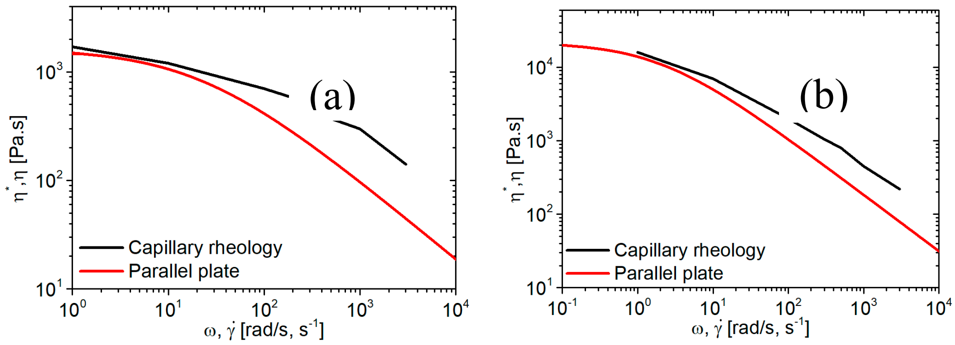

Figure S3. Parallel plate and capillary rheology data for PS: (**a**) PS0S, (**b**) PS2S at 200 °C

3. Continuum models.

3.1. Wang-Hill Model

Wang and Hill postulated a layer of thickness δ on the order of *dt* surrounding each nanoparticle, where the viscosity in the layer (η_{in}) is equivalent to the Rouse viscosity, i.e. $\eta_{in} = \eta_{Rouse}$. The η_{Rouse} is the viscosity calculated by the Rouse model and the bulk viscosity (η_{out}) is given by the reptation model, i.e. $\eta_{out} = \eta_{rep}$. They also accounted for finite slip at the particle surface by means of a reciprocal slipping length, *k*. Where $k \rightarrow \infty$ corresponds to no slip, whereas $k = 0$ corresponds to complete slip of the polymers along the nanoparticle surface. These conditions are used to solve the Stokes equation focusing on very low shear rates ($\dot{\gamma}$), i.e. 0.01 s^{-1}, for nanoparticle radius $r_{particle} \approx 7.5$ nm, and radius of gyration of the polymer $R_g \approx 7$ nm. This is in good agreement with the experimental parameters set in our experiments. It is worth mentioning here that this model does not account for particle-particle interaction. The viscosity change given by the model of Wang and Hill is given by:

$$[\eta] = \frac{(A'\chi 2 + B'\chi + C') + k\, r_{particle}\, (D'\chi 2 + E'\chi + F')}{(A\chi 2 + B\chi + C) + k\, r_{particle}(D\chi 2 + E\chi + F)} \quad (1)$$

where $\chi = (\eta_{in}/\eta_{out})/(\varrho_{in}/\varrho_{out})$ and in and out correspond to the Rouse layer around the particle and the bulk, respectively. The definitions for A'–F' and A–F can be found in reference [2] and are related to the radius of the nanoparticle ($r_{particle}$) and to the layer thickness δ. The viscosity change [η] for each filler volume fraction (φ) is found as a function of δ, $\eta\, in/\eta\, bulk$, and k using the continuous mass flux boundary condition ϱ in = ϱ out. This leaves us with four parameters: the thickness (δ), radius of the nanoparticle, the Rouse viscosity (η_{in}) and the bulk viscosity (η_{out}). The radius of the nanoparticle is known from the TEM measurements, while the Rouse viscosity is calculated using the relation $\eta_{rouse} = \eta_{in} = \eta_c \left(\frac{M}{M_c}\right) a_T$ where M_c and η_c are the critical molar mass and viscosity, respectively and a_T is the shift factor. The M_c = 5000 g/mol for PC_{30}. The melt viscosity or the bulk viscosity is calculated using relation: $\eta_0 = \eta_{bulk} = \eta_{out} = \eta_c \left(\frac{M}{M_c}\right)^a a_T^{13}$ using $a_T = 1$ at 250 °C, the ratio of $\frac{\eta_{in}}{\eta_{out}}$ is given by $\chi = \frac{\eta_{in}}{\eta_{out}} = \left(\frac{M}{M_c}\right)^{1-a}$ where a = 3.4 for linear PC (PC_{30}) and 5.14 for branched PC (PC_{40}). For PC_{30} χ = 0.0136 and PC_{40} χ = 0.0001824.

4. SHIFT FACTORS

4.1. PC:

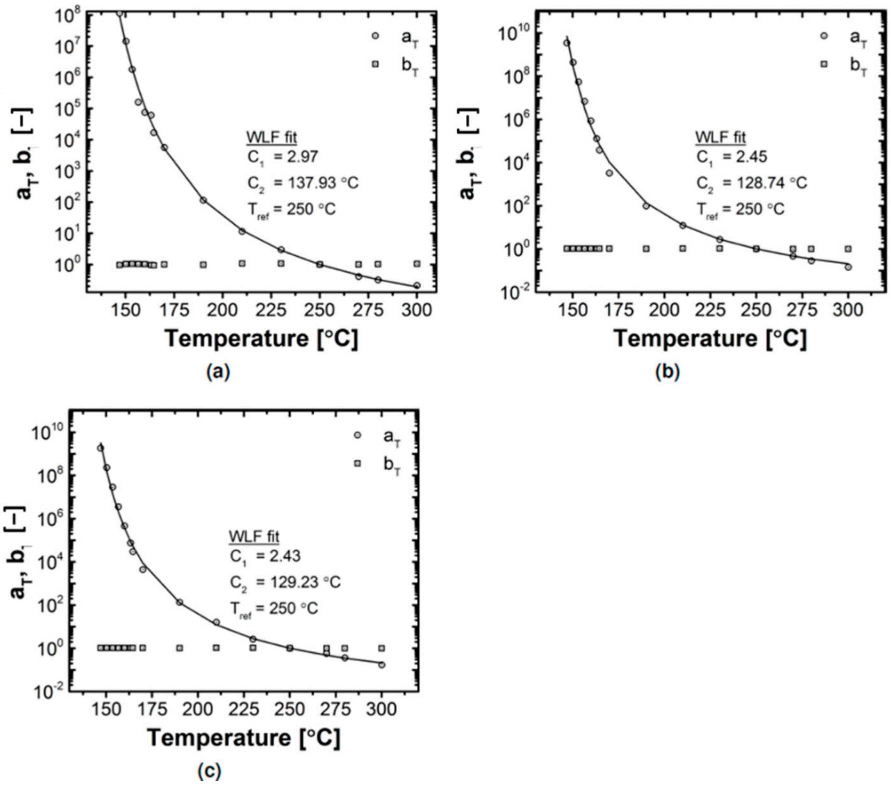

Figure S4. Shift factors employed in obtaining TTS master curves for PC: (**a**) PC_{30}, (**b**) PC_{30}0.7S, and (**c**) PC_{30}1.5S. The solid line is the WLF equation fit.

4.2. PS:

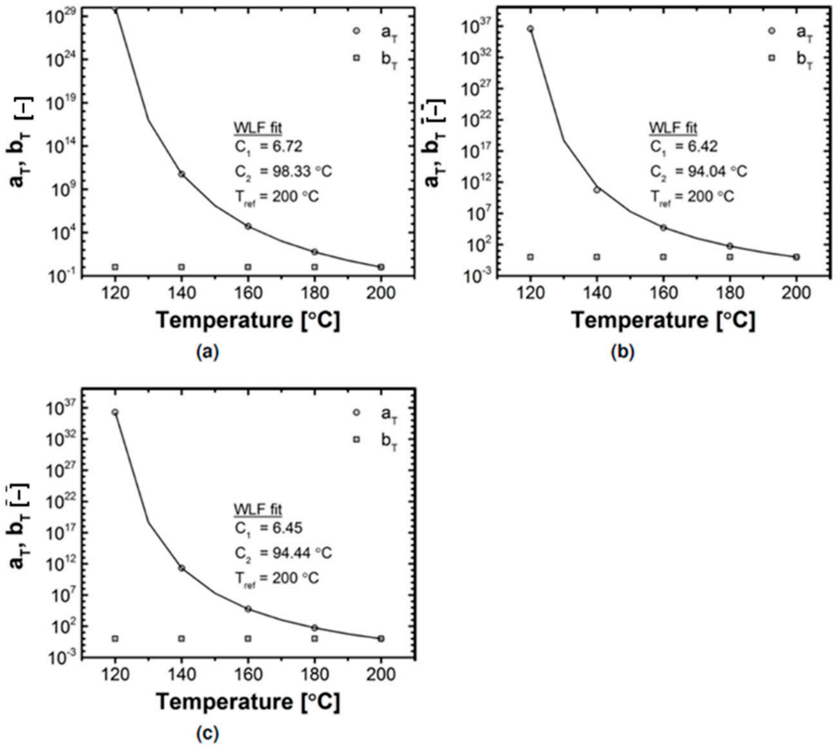

Figure S5. Shift factors employed in obtaining TTS master curves for PS: (**a**) PS0S, (**b**) PS1S, and (**c**) PS2S. The solid line is the WLF equation fit.

Reference

1. Henson, D. J.; Mackay, M. E., Effect of gap on the viscosity of monodisperse polystyrene melts: slip effects. *Journal of Rheology* **1995,** *39* (2), 359–373. doi:10.1122/1.550702
2. Wang, M.; Hill, R. J., Anomalous bulk viscosity of polymer-nanocomposite melts. *Soft Matter* **2009,** *5*, 3940–3953. doi:10.1039/B905686F
